# A multi-source entity-level sentiment corpus for the financial domain: the FinLin corpus

**DOI:** 10.1007/s10579-021-09555-3

**Published:** 2021-08-16

**Authors:** Tobias Daudert

**Affiliations:** 1 Insight SFI Research Centre for Data Analytics, Data Science Institute, National University of Ireland, Galway, Ireland; 2 Lower Dangan, Newcastle Galway City, H91 AEX4 Ireland

**Keywords:** Corpus, Sentiment, Finance, Microblogs, News, Reports

## Abstract

We introduce FinLin, a novel corpus containing investor reports, company reports, news articles, and microblogs from StockTwits, targeting multiple entities stemming from the automobile industry and covering a 3-month period. FinLin was annotated with a sentiment score and a relevance score in the range [− 1.0, 1.0] and [0.0, 1.0], respectively. The annotations also include the text spans selected for the sentiment, thus, providing additional insight into the annotators’ reasoning. Overall, FinLin aims to complement the current knowledge by providing a novel and publicly available financial sentiment corpus and to foster research on the topic of financial sentiment analysis and potential applications in behavioural science.

**Supplementary Information:**

The online version contains supplementary material available at 10.1007/s10579-021-09555-3.

## Introduction

Modern societies and their welfare rely on market economies making millions of people around the globe affected by changes in the markets (Nassirtoussi et al., 2014). It is fundamental to understand what influences the markets, particularly the financial markets as a proxy for the market economies, as well as how they react and how strong is this influence. Over the last 20 years, sentiment analysis aiming at measuring public mood in respect of the financial domain has become an important field of research. Sentiments are extracted from various data sources, one example is news articles. Within this source, one can find discussions regarding macroeconomic factors, company-specific reports or political information, all of which can be relevant to the markets (Sinha 2014). As most of the current research is based on specific news such as financial or corporate, it can be fruitful to combine information on textual sentiment stemming from other publicly available sources such as blogs and company reports. Only a few researchers (Rachlin et al., 2007; Schumaker & Chen 2009; Gao & Japkowicz 2009; Schumaker et al., 2012; Hagenau et al., 2013) have experimented with such a hybrid approach. However, a dataset combining multiple sources while covering the same period for financial textual sentiment is absent. Such a dataset comes with many potential applications which remain unexplored to this date, examples are the effect of sentiment contagion (Shi et al., 2019), leveraging contextual sentiments which is especially beneficial to enhance the detection of implicit sentiments, and development of improved market sentiment indices. All these research topics require sentiment information based on a broad audience, stemming from different data types and sources, annotated on a scale, covering a set of sentiment targets, and stemming from the same period.

To complement the current knowledge, we present FinLin, a novel and publicly available financial sentiment corpus consisting of four contemporaneous data types: news, microblogs, company reports, and investor reports. While consisting of multiple data types, it covers the same period and targets the same set of entities across all sources, thus, linking records through a set of shared entities. In addition, FinLin is annotated with the novel concept *relevance*. This concept is designed to enable the detection of sentiment contagion and provide a means to improve methods aggregating multiple sentiments for the creation of sentiment indices.

## Related work

Sentiment analysis is currently a trending research topic in natural language processing (NLP). While its technical goal is to analyse peoples’ sentiments towards entities and their attributes in written text, its applications are widespread and are already affecting our daily lives; the detection of fake reviews, the study/survey of peoples’ political opinion, or automatic stock trading based on Twitter data, are a few examples (Liu, 2012). Although the term was only coined in 2003 (Liu, 2012; Nasukawa & Yi 2003), research on sentiment analysis has been very active since 2000 (Wiebe, 2000; Liu, 2012). Opinion mining is commonly used synonymous to sentiment analysis, however, it is still debatable whether these are distinct terms. Under the umbrella of opinion mining, even more tasks are attributed to this field such as emotion mining, suggestion mining, or subjectivity analysis.

Textual information can be classified into facts and opinions (Liu et al., 2010). While facts are “objective expressions”, opinions are “subjective expressions that describe people’s sentiments, appraisals or feelings” (Liu et al., 2010). In the financial domain, seldom works deal with sentiment analysis following the definition by Liu et al. (2010). This occurs because most financial texts are not subjective. For example, news articles tend to be factual (Van de Kauter et al., 2015); similarly company reports usually provide factual information on a company rather than the management’s feelings, or follow legally required templates. According to the definition by Liu et al. (2010), non-subjective texts should be labelled as neutral; however, in the finance domain, these texts still bring relevant information to a variety of tasks. For example, Schumaker et al. (2012) classify texts into subjective, objective, and neutral, with a second-level classification of the subjective texts into positive, negative and neutral. The work of Jin et al. (2019) defines financial sentiment analysis as the classification into bearish and bullish. Consequently, the goal is not to analyse the authors sentiment but to find whether the author believes the discussed asset will increase (i.e. the author is bullish), or decrease (i.e. the author is bearish) in price. In addition, texts can contain implicit sentiments - a text fragment which expresses subjective sentiment without containing an explicit sentiment word (Liao et al., 2019). While texts containing implicit sentiments are opinionated, they are often difficult to identify, especially in a financial or economic setting where a person requires background knowledge and reasoning to make a decision. The sentence “Wow they increased their sales by 2% [...]” can be negative if the referred asset had previously increased sales in the double-digits. While “Wow” can indicate an opinion, the implicit sentiment is not obvious.

Initial works on sentiment analysis mainly relied on lexicons (Hu & Liu 2004; Kim & Hovy 2004; Nigam & Hurst 2004; Ding et al., 2008), such as WordNet, while currently, machine learning (ML) and deep learning (DL) techniques are the preferred approach (Do et al., 2019). While semi-supervised approaches also exist, ML and DL approaches can be broadly classified into supervised, and unsupervised, with the first requiring manually labelled training data which can be labour intensive and costly to obtain. Unsupervised tasks offer an inexpensive alternative, nonetheless, it comes with some caveats, namely the need for large-scale datasets which are difficult to acquire, especially when dealing with multiple data sources. Hence, this leads to the growing need for labelled datasets for sentiment analysis adapted to different domains and environments. It is also important to highlight the necessity of data covering a variety of languages; it has to be accounted for that labels might not be accurate in different settings. For example, *lush* can describe an area with “a lot of green, healthy plants” in American English while in British English, it can also mean “a person who regularly drinks too much alcohol”, although sharing the same language (Cambridge Dictionary, 2011). Semantic change (Grzega & Schoener 2007) plays its role, for example, *awful* originated as a positive term while currently it is used with a negative connotation (Wijaya & Yeniterzi 2011).

Given the many challenges in sentiment analysis, a myriad corpora exist. As such, we highlight a selection of recent corpora:

- **IMDB corpus** Contains 25,000 textual movie reviews from IMDB^1^ annotated as positive and negative (Maas et al., 2011).
- **Sentiment stanford sentiment treebank** Comprises reviews from the entertainment review website Rotten Tomatoes^2^ annotated using a sentiment scale between 1 and 25 (Socher et al., 2013).
- **Sentiment140** Consists of 160,000 tweets annotated in polarities (positive, neutral, negative) after removing emoticons (Go et al., 2009).
- **SemEval2017 Task 4** One sub-task deals with polarity detection in tweets in English and Arabic, while another deals with a 5-point scale sentiment classification (Rosenthal et al., 2017).
- **SemEval2017 Task 5** Addresses fine-grained sentiment analysis for microblogs (Twitter and StockTwits) and news annotated with a fine-grained sentiment scale between − 1 and 1 (Cortis et al., 2017).
- **The SSIX Corpora** Presents three financial corpora consisting of StockTwits and Twitter messages in English, German, and Spanish. Similarly to the SemEval2017 Task 5, the sentiment is annotated with a fine-grained scale between − 1 and 1 (Gaillat et al., 2018).
- **FiQA 2018 Challenge** Addresses aspect-based sentiment analysis for text instances in the financial domain using microblog and news headlines. The sentiment is also annotated with a fine-grained scale between − 1 and 1 (Maia et al., 2018).

Overall, corpora can consider one type (e.g. microblogs) or one source of data (e.g. Twitter), as well as being open-domain (e.g. Sentiment140) or domain-specific (e.g. SemEval2017 Task 5). In the case of the financial domain, previous works have focused on stock price prediction and on unsupervised sentiment analysis. Bollen et al. (2011) uses Twitter microblogs together with the OpinionFinder sentiment lexicon^3^ to measure correlations between mood in tweets and the stock prices. In a similar fashion, Lee et al. (2014) aim at the stock price prediction using 8k reports, mandatory for U.S. publicly listed companies, together with SentiWordNet.^4^ Li et al. (2014) use 5 years of FINET^5^ stock news in the English language and apply the McDonald financial sentiment dictionary (Loughran & McDonald 2011), as well as the Harvard IV-4 sentiment dictionary to identify sentiments.^6^ The SemEval 2017 Task 5 was the first labelled sentiment analysis corpus dealing with fine-grained sentiment, in the financial domain. It consisted of two sub-tasks, the first was focused on microblogs while the second dealt only with news data. However, the microblogs and news do not cover the same period nor entities, thus, its use is limited as it cannot be applied in tasks requiring contemporaneous data. Following works such as the SSIX and FiQA corpora carry the same limitations, although including data in different languages they do no cover the same entities across multiple data types in a delimited time span. This issue was faced in our recent work (Daudert et al., 2018; Daudert & Buitelaar 2018); to obtain news contemporaneous to the microblog data, we faced several limitations as the news collection was performed almost 2 years after the publication of the SemEval dataset and resulted in a medium-sized dataset.

**Fig. 1:**
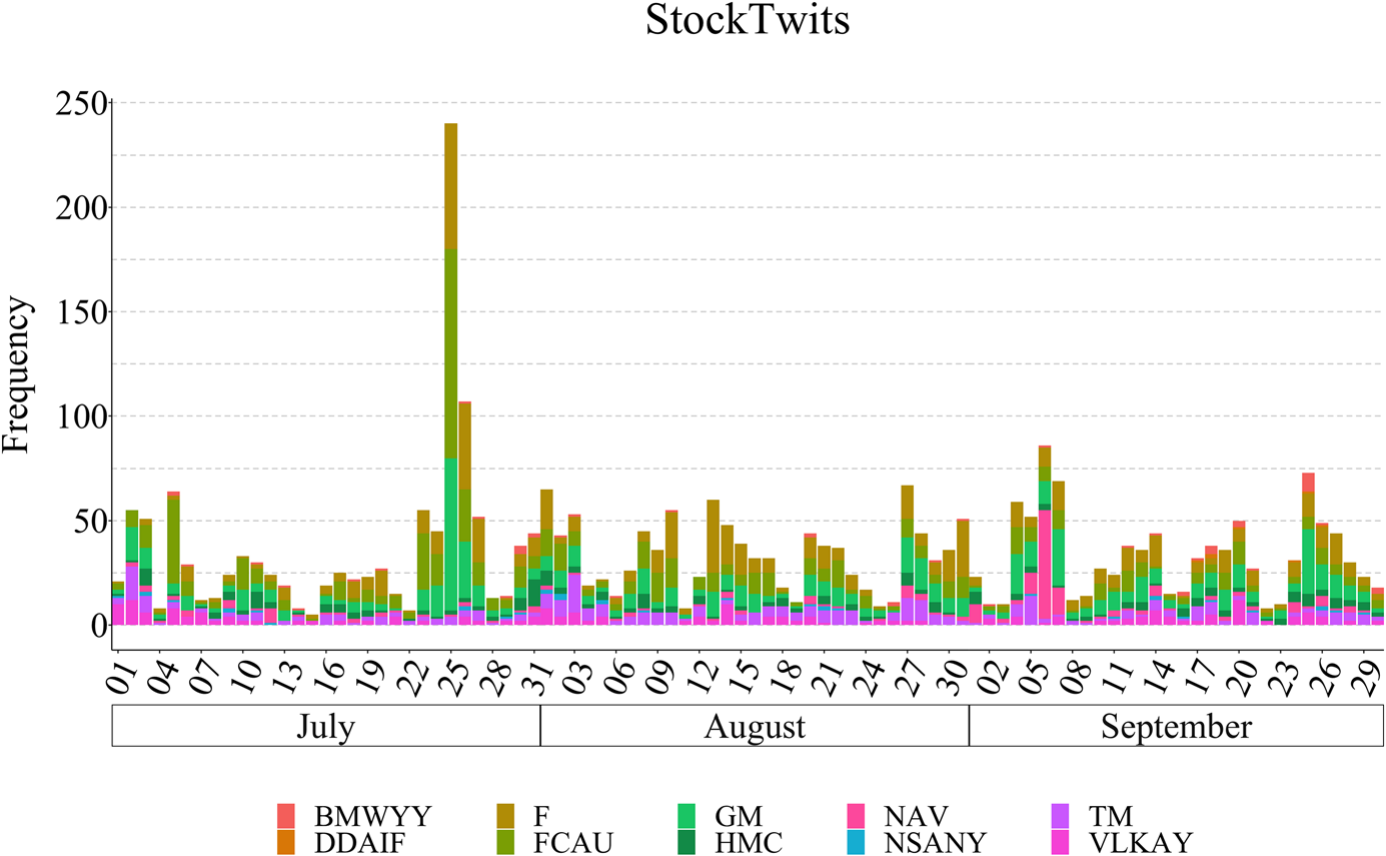
Distribution of the stockwits in FinLin. The stocktwits are aggregated according to the tickers specified in Table 1. The * x* axis is labelled with a 3-day scale

**Fig. 2:**
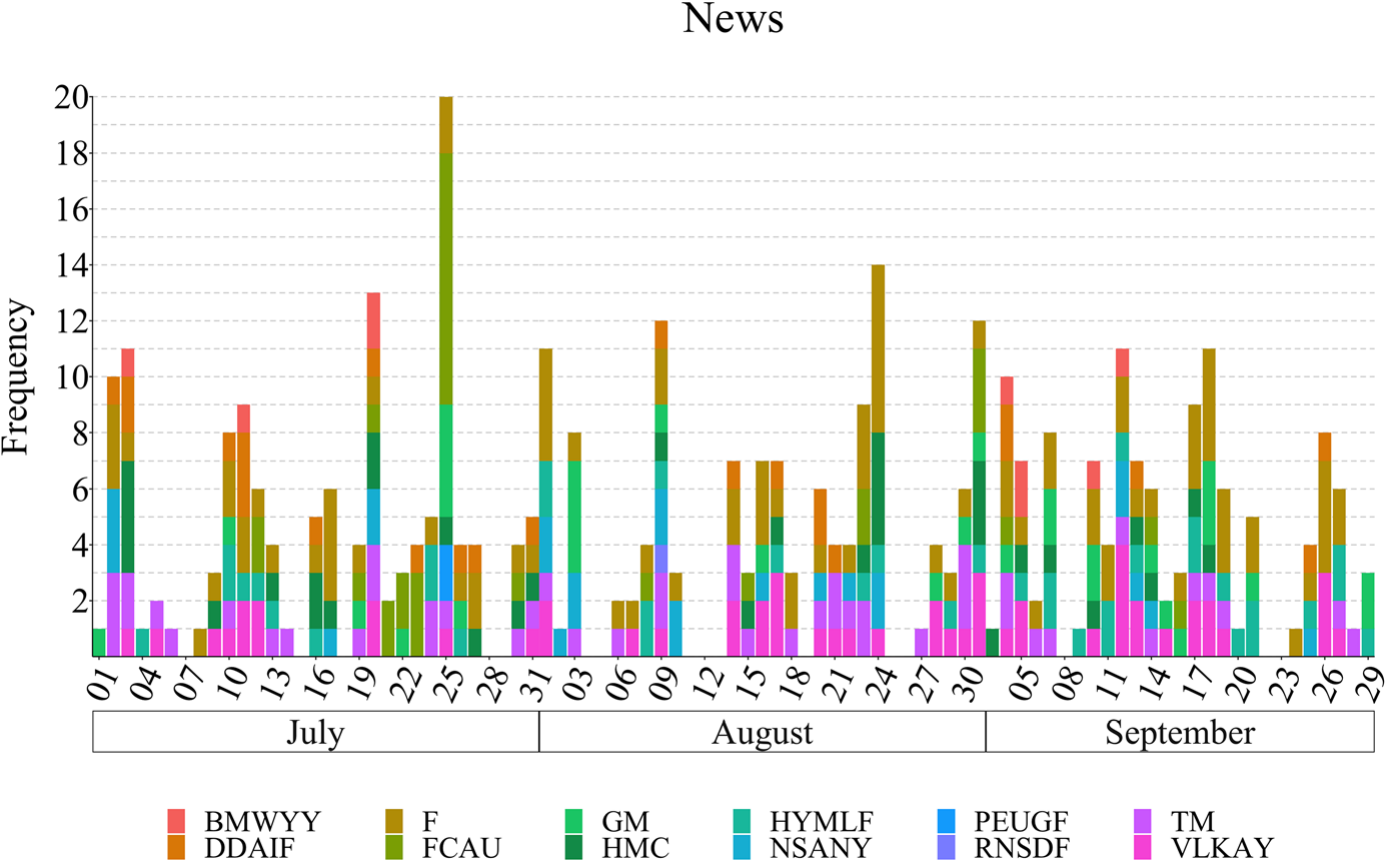
News distribution in FinLin. The news are aggregated based on the tickers present in Table 1. The* x* axis is labelled with a 3-day scale

With the identified gaps in mind, as well as the requirements needed for further advancements in the field, we develop and release the novel FinLin dataset. This labelled dataset covers multiple data sources and types (i.e. microblogs, news, investor, and company reports), the same period, and the same entities (Figs. 1, 2).

## Corpus design

The aim behind FinLin is to enable the exploration of sentiments directed at targets across different data sources in a financial setting. Throughout this paper, we utilise the terms *target*, *entity*, and *company* interchangeably to represent the sentiment target i.e. the companies defined in Sect. 3.1.

To select the data sources covered by FinLin, we rely on past research to choose four distinct sources representing three data types: microblogs, news articles, and reports. While *data source* refers to the origin of the data, *data type* classifies it into general categories based on their source and attributes. Based on the available labelled sentiment datasets for finance (Cortis et al., 2017; Gaillat et al., 2018; Maia et al., 2018), we include StockTwits as the microblog source. Our previous works (Daudert et al., 2018; Daudert & Buitelaar 2018) indicated a significant relation between news and stocktwits, hence, we also consider news articles. Besides, we include analyst reports and company reports to capture a professional view and first-hand information on a company. In addition, these reports have shown to have a significant impact on the markets and have been targeted in past research (Li et al., 2006; Sinha, 2016). For date-time compatibility, the data collection occurred simultaneously on all sources during the year 2018. To ensure matching information, we defined a set of targets stemming from the automobile sector, beforehand. The data was then annotated and consolidated by domain experts. The following sections detail the sources and entities considered, the information collected, as well as the annotation process.

### Entities

For this dataset, we consider data regarding the automobile sector as it is currently facing a phase of changes (e.g. electric cars, CO_2_ emissions regulations, self-driving cars) with several stakeholders (e.g. Volkswagen, Toyota, Ford), hence, receiving wide coverage. We initially limit the data collection to the worldwide top 20 car manufacturers based on the number of produced vehicles as published by the *Organisation Internationale des Constructeurs d’Automobiles* (OICA)^7^.

**Table 1:** Automobile companies, their nicknames, cashtags, ticker, as well as subsidiaries and their brands

Company name	Nickname	Cashtags	Ticker	Other subsidiaries
Toyota motor Corp	Toyota	$TM	TM	Hino, Lexus
Volkswagen AG	VW	$VLKAY	VLKAF VLKAY POAHF	Audi, Bentley, Bugatti, Ducati, Lamborghini, MAN, Porsche, Scania, (Seat), Skoda
Hyundai motor Co	Hyundai	–	HYMLF HYMTF KIMTF	Genesis, Kia
General motors Company	G.M.	$GM	GM	Buick, Chevrolet, Holden
Ford motor Co	Ford	$FORD	F	Cadillac, Lincoln
Nissan motor Co	Nissan	$NSANY	NSANY	Datsun, (Infiniti)
Honda motor Co	Honda	$HMC	HMC	Acura
Fiat chrysler automobiles NV	Fiat	$FCAU $RACE $FERI	FCAU	Abarth, Alfa Romeo, Chrysler, Dodge, Lancia, Maserati
Renault SA	Renault	–	RNSDF, RNSLY	Dacia, Lada
Peugeot SA	Peugeot	$UG	PEUGF	Citroen, Opel, Vauxhall
Suzuki motor corp	Suzuki	$SZKMY	SZKMY	–
Navistar	–	$NAV	NAV	–
Daimler AG	Daimler, Mercedes, Mercedes-Benz	$DDAIF	DDAIF	Smart
Bayerische motoren werke AG	B.M.W.	$BMW	BMWYY	Mini Cooper

Subsidiary companies in brackets were not considered due to their high ambiguity

Given our focus on the English language, a portion of the mentioned companies have a reduced representation or are absent in the four distinct sources considered. Thus, to avoid data sparseness of certain entities in FinLin we excluded the following companies: SAIC Motor Corporation Limited, Suzuki K.K., Geely Automobile Holdings Ltd, Chongqing Changan Automobile Co Ltd, Mazda Motor Corporation, Dongfeng Motor Group Co Ltd, BAIC Motor Corporation Ltd, Mitsubishi Motors Corporation. To track the remaining, we also shortlisted the respective brand names and subsidiary companies; the final list of selected companies is shown in Table 1. Texts referring to Porsche and Kia were also collected using the tickers POAHF and KIMTF, respectively; to present the results we aggregate these accordingly to the parent company i.e. Volkswagen and Hyundai.

### Sources

#### Microblogs

The rapid expansion of social media has changed how people communicate and express their opinions making it one of the main sources of public sentiment. Founded in 2008, StockTwits^8^ is a microblog platform tailored to investors, with over 2 million users currently registered.^9^ In a similar fashion to Twitter, users share short messages referred to as stocktwit(s). However, a particular characteristic of a stocktwit is the addition of *cashtags*. A cashtag corresponds to any Stock, Future, or Forex ticker prefixed with the $ symbol; for example, Toyota Motor Corporation is represented by the $TM cashtag. This enables the aggregation of all information targeting a determined target. The collection of stocktwits was performed using the StockTwit Application Programming Interface (API)^10^ utilising the selected company names and respective chastags.

#### News

News articles, written work which is published in either a print or electronic medium, have been shown important for stock price prediction and the financial markets by proxy. However, as the amount of news articles is continuously increasing, machine-based systems are crucial to filter noise (Li et al., 2014). News articles can also be long dealing with one or many events which are put into context. News were retrieved from Yahoo! News^11^; as it is aggregating articles from multiple providers, this platform provides a simple and convenient way to cover several newspapers. Yahoo! News was accessed daily to find and store all news articles containing at least one of the terms shown in Table 1 in the headline.

#### Company reports

Company reports reflect companies’ financial performance and strategy, and typically include quantitative data (accounting and financial data) and qualitative data (narrative texts). As proposed by Hájek and Olej (2013), these reports also describe the managerial priorities of a company, hence, they tend to differ in terms of the subjects emphasized when the company’s performance worsens (Kohut & Segars 1992). For this corpus, we also chose to focus on company reports since they provide first-hand information by company officials and play a crucial role when it comes to assessing a company’s performance. As there is no automated solution for the retrieval of reports, we manually gathered the publicly available company reports from the respective company’s website which usually come in a PDF format.

#### Investor reports

Investor reports, also called analyst reports, provide information about an analyst’s assessment of a company and its future performance. Different from opinions on StockTwits, this type of reports provides detailed reasoning and is often done by professionals who would closely follow a group of companies for multiple years. The investor reports were collected from Seeking Alpha^12^, a crowd-sourced content service for financial markets with investors and industry experts as contributors.

**Fig. 3:**
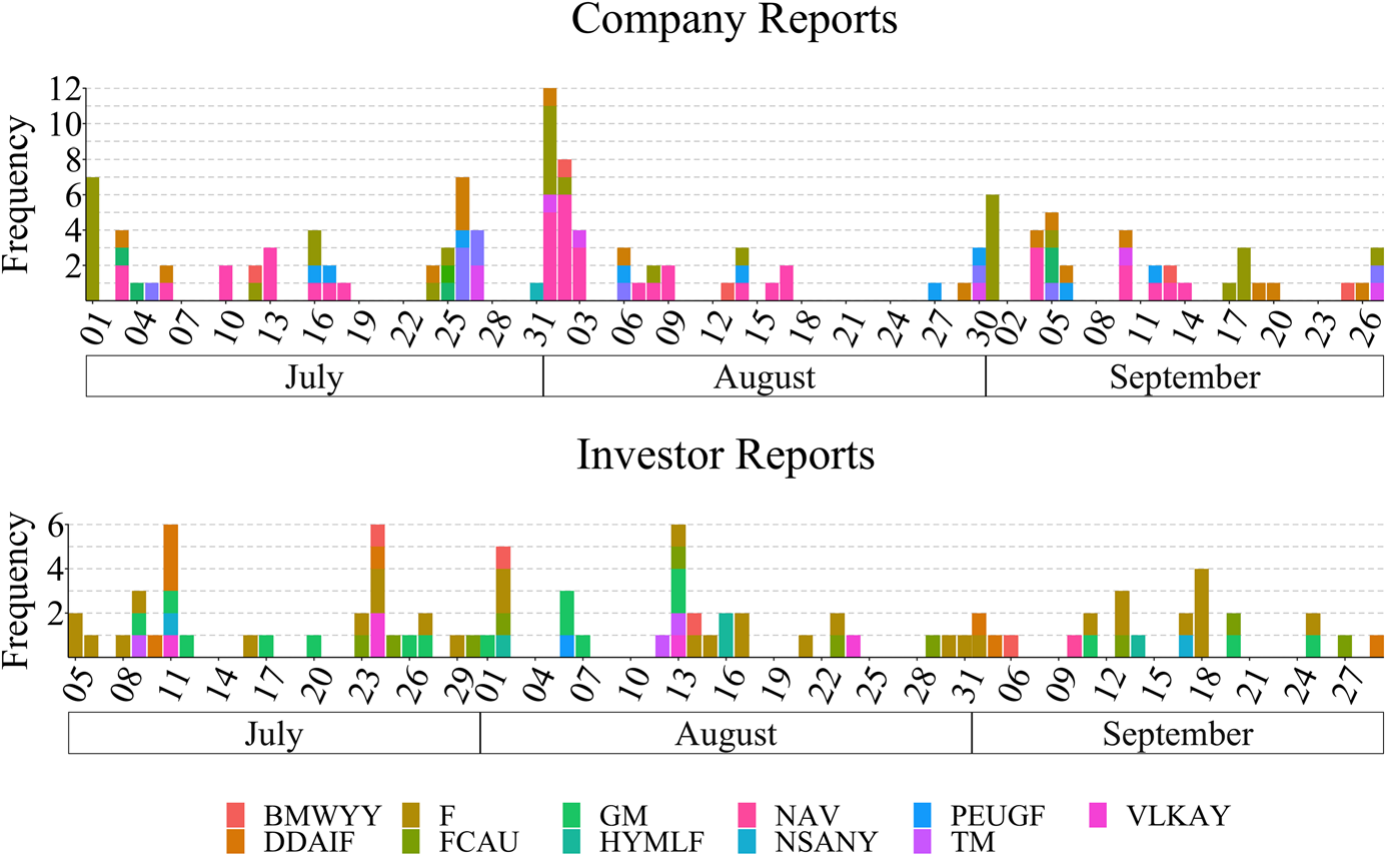
Distribution of all reports per target in FinLin. The reports are aggregated according to the tickers specified in Table 1. The* x* axis is labelled with a 3-day scale

#### Timeframe selection

We initially collected data for the entire year 2018, for all targets and sources. Given the available budget for the data annotation (see Sect. 4.5), we chose a period of 3 months matching the common standard of quarterly reporting, hence, ensuring the occurrence of company reports within this timeframe. We specifically chose the period of 01/July/2018 to 30/September/2018 based on the data diversity and its volume which is highest in the 3rd quarter of 2018. Figure 3 shows the frequency distribution for the investor and company reports; Figs. 1 and 2 present the distribution for the news and stocktwits, respectively.

## Corpus annotation

Given our selected data covering a period of 3 months, the shortlisted entities and their subsidiaries, we conduct the following pre-processing steps in preparation for the annotation task. Specifically, we conduct down-sampling for the stocktwits, sentence extraction for the news articles and investor reports, and a manual text extraction for the company reports.

### Data preparation

#### Stocktwits down-sampling

The initial data collection for the specified period yield 13,243 stocktwits; however, given that 91.7% of the stocktwits deal with the entities F, GM, and FCAU, we chose to limit the maximum number of stocktwits to 700 per entity to avoid data over-representation and due to monetary constraints. We apply a random equal probability sampling algorithm, from the Pandas library^13^, to obtain the final dataset consisting of 3204 stocktwits.

#### Sentence extraction for news and investor reports

Given the nature of news articles and investor reports, consisting of longer descriptive and contextualised texts, we decide for the annotation of text portions certainly dealing with the entity of interest. During initial exploration of the collected news and reports, we noticed a shared behaviour: the articles’ core information either appears at the beginning of a section dealing with the tracked entity or in the next few sentences. Some investor reports even deal with multiple entities at the same time, thus, not all of the text is important to our analysis. Hence, we extract the sentence in which the entity occurs first and the following two sentences. The extracted three sentences and the title of the news/report are then subject to the annotation task. Note that there are also cases in which other entities are mentioned in the content but do not constitute the main article topic. Therefore, we focus on annotating entities specifically named in the headline of a news article.

#### Text extraction for company reports

Company reports are usually provided in PDF format, as such, we had to manually extract their content. Although tools for the automatic extraction of PDF content exist, we decided to conduct a manual text extraction based on two reasons: Firstly, as the analysis goal of this dataset is of a qualitative nature, extraction issues could severely affect the results; secondly, as we are dealing with a limited number of reports, small extraction errors could lead to contrasting differences in the final statistics. Furthermore, the dataset’s reasonable size allows for undemanding manual extraction.

**Fig. 4:**
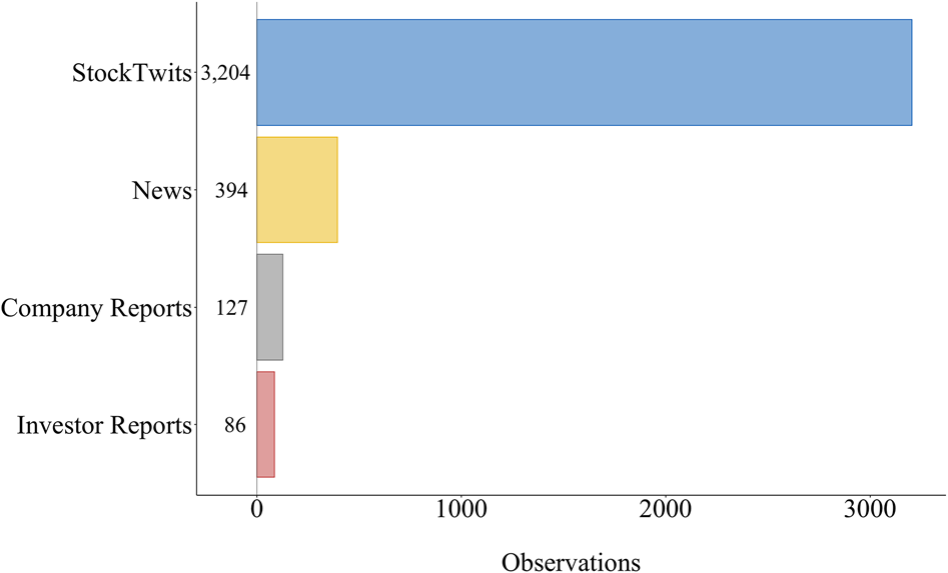
Number of observations for each data source

**Fig. 5:**
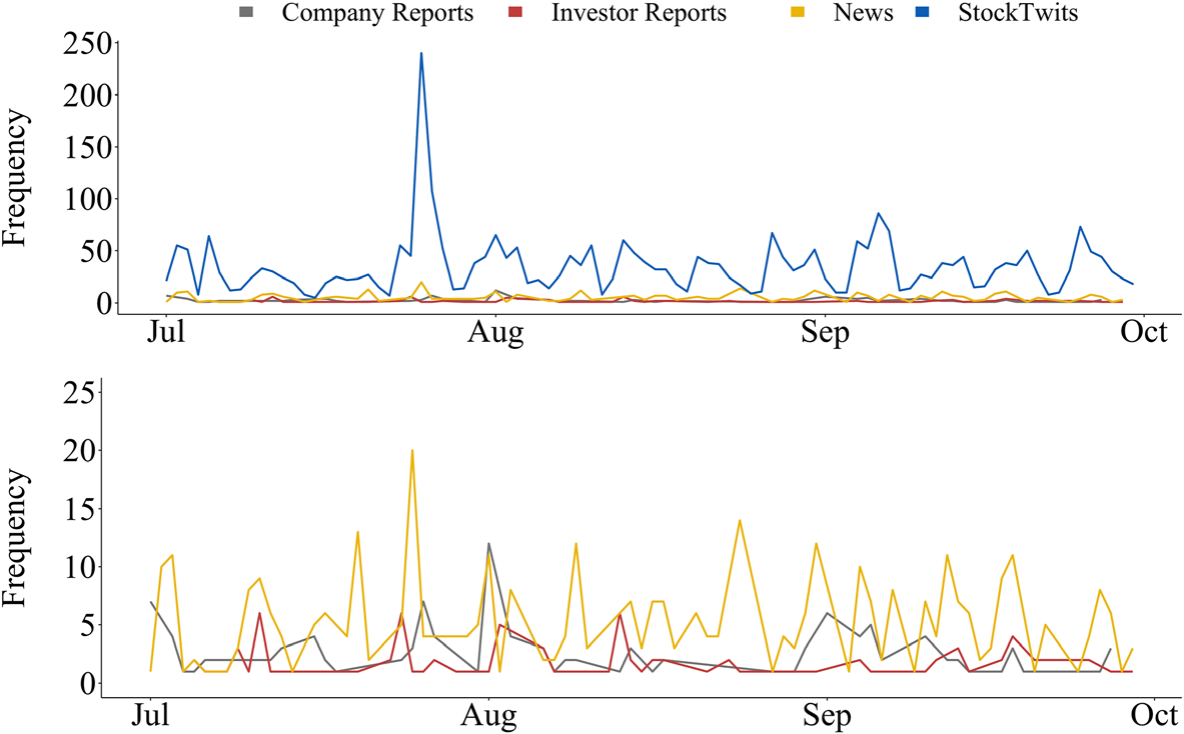
Time series distribution for sources in the FinLin corpus. The bottom plot shows the distribution for the Company Reports, Investor Reports, and News, using a smaller * y*-axis

The total number of investor reports, company reports, news, and stocktwits are presented in Fig. 4 and the respective time-series in Fig. 5. Note that, in these figures, we report on the number of entities per news article; as previously stated the same news article can be annotated for multiple entities.

### Point of view

**Fig. 6:**
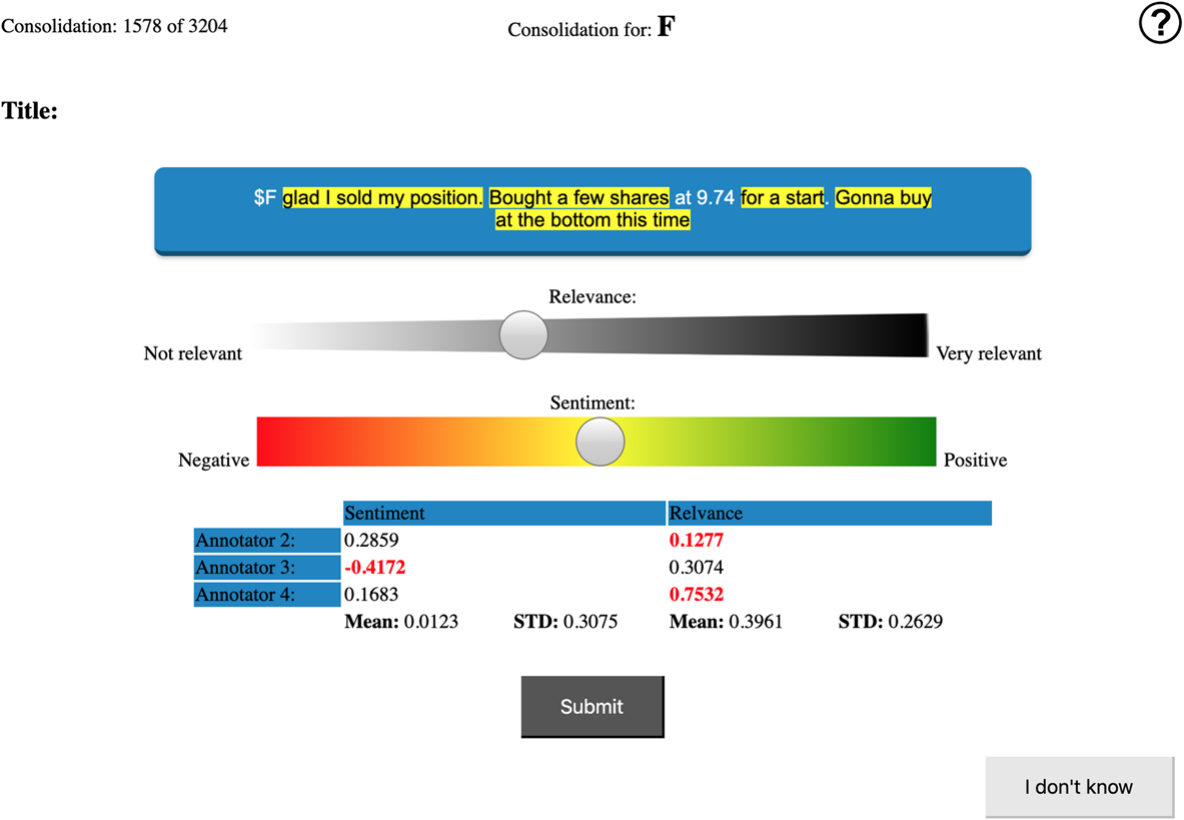
Close-up of the consolidation mode in the applied tool

One of the main challenges when understanding, interpreting, and annotating texts for sentiments corresponds to the correct *point of view*. According to Liu (2012) the definition of *subjective sentiment* can be formalised as a quadruple,

1 $$\begin{aligned} (g, s, h, t) \end{aligned}$$

where *g* corresponds to the sentiment target, *s* the sentiment about the target, *h* the sentiment holder, and *t* the time of expression. Applying this formalisation to define the *point of view*, an annotator can interpret a text from the point of view of *h*, or *g*. While the first matches the common understanding of a (subjective) sentiment (i.e. how does the author feel), the latter is especially important in a financial sentiment setting (i.e. what is felt about the target). When analysing financial texts, researchers want to know what is the sentiment directed at a given entity, rather than knowing what the author feels towards the given entity (Li et al., 2014; Jin et al., 2019). Taking the example sentence in Fig. 6, the annotator can interpret the text from the *point of view* of the author *h* “glad I sold my position [...]” (i.e. what the author feels towards Ford.), or from the *point of view* of Ford ($F) (i.e. what the selling of the author’s position implies for Ford.). As one of the dataset aims is to support financial analysis on the selected automobile companies, the *point of view* refers to the presented target, using the previous example, *g* corresponds to Ford.

### Factual and subjective sentiment

As previously addressed, FinLin contains data types, namely news and reports, which naturally do not contain opinionated information. While Liu et al. (2010) defines *subjectivity* as a requirement for sentiment, the financial domain (see Sect. 2) is not restricted to the analysis of the sentiment holders but rather interested in the sentiments directed at targets. Given an objective expression, sentiment refers to an assessment of the presented information given the desired *point of view*. These expressions are named *factual sentiments*. Therefore, FinLin incorporates *factual* as well as *subjective* sentiments, but always from the point of view of the specified target. A *factual sentiment* is a sentiment based on underlying facts which can occur, for example, in a news article (e.g. “the company is expected to increase its sales”) which itself is objective and does not reveal any connotation about the author’s feelings regarding this information. A *subjective sentiment* is a sentiment which comes with a subjective evaluation of a piece of information, or reveals what an author thinks or feels e.g. “glad I sold my position”, “I like that the company is expected to increase its sales”. This is a strong difference to the existing sentiment datasets which only annotate the author’s (subjective) sentiment. Extending the definition of sentiment by Liu (2012), mentioned in the previous subsection, the formal representation of a *factual sentiment* is defined as a quintuple,

2 $$\begin{aligned} (g, f, s, h, t) \end{aligned}$$

where *f* corresponds to the sentiment forming fact and *s* now reflects the sentiment about the target, based on the fact. Comparing quadruples 1 and 2, we can observe that a factual sentiment is based on an explicit fact *f* while the subjective sentiment can lack reasoning. Given the ambiguity of factual expressions with ones containing an implicit sentiment, *f* enables the annotator to infer *s*.

Looking at a trading context, positive sentiment is often seen equal to *bullish* and negative as *bearish* (*i.e.* buy/long or sell/short).^14^ Given the point of view of the target entities, this is in line with our definition of factual sentiment as well as subjective sentiment. Figure 6 again shows an intricate example: “glad I sold my position. Bought a few shares at 9.74 for a start. Gonna buy at the bottom this time”. On the one hand, the author expresses their subjective sentiment “glad I [...]” and on the other hand, they reveal a bearish factual sentiment “Gonna buy at the bottom this time” which implies the future expectation of the target Ford (F) reaching the bottom (i.e. decreasing in value). Based on the *point of view* (Ford), the sentiment is expressed in the latter extract i.e. bearish sentiment. An investor can have a positive (subjective) sentiment regarding a company if they are invested short meaning they expect a decreasing company performance or stock price while this is not good for the company itself and implies a bearish (trading) sentiment. Contrary, they can have a negative (subjective) sentiment if invested short on a value-increasing (bullish) stock. Therefore, positiveness and negativeness always depend on the point of view.

### Relevance annotation

The previous two subsections can be summarized as follows: texts can be analysed according to the sentiment holder (Case 1), or the sentiment target (Case 2). Sentiments can be subjective if expressed by the sentiment holder (Case 3) or factual (Case 4); Case 4 can affect objective instances, or instances with implicit sentiment.

Given that financial sentiment analysis is usually interested in the sentiment towards a target (i.e. Case 2), this leads to the consideration of multiple sentiments. For example, if five texts targeting Ford are published in a period *p*, the sentiment for the target is an aggregate of the five texts. Given that all texts are not equally important for this aggregate sentiment (e.g. some are more credible, or some are authored by an influential person), it is mandatory to determine the strength of a text’s influence and how it could affect the aggregate sentiment.

To quantify this influence, we consider Case 3 and Case 4. In the former, when a fact is absent, the reputation of the author can influence the impact of the authored texts as well as its perceived credibility (i.e. the way a text is formulated; coherent structure, no logical gaps, appropriate wording). In Case 4, the authorship can be secondary, however, the text’s perceived credibility also matters, as well as, first of all, the presented fact. For example, facts directly affecting the business can be more influential than facts related to the company’s CEO. We name this potential influence of a text on the aggregate (i.e. market) sentiment **relevance**. While the *sentiment* measures one author’s sentiment directed at the target, the *relevance* measures a sentiment’s potential influence (relative to other sentiments) on the target’s aggregate sentiment.

### Annotator selection

As FinLin refers to a specific domain, we employed a process to find and select annotators with knowledge in finance and stock trading. The process began with the task advertisement in social media, university channels as well as the local School of Business and Economics. In total, 26 people responded to the advertisement; upon surveying the respective *Curriculum Vitae* we selected 12 people given the criteria:

1. Higher education in Finance, Business, or Economics;
2. Professional experience related to Finance, Business, or Economics;
3. Stock trading experience;
4. English native.

These potential annotators were then invited to a selection test in which they were tasked to annotate 100 texts. Given the quality of the annotations (reviewed by the author), the time needed for each annotation, and the ability to work with the annotation interface, the final selection corresponded to three annotators and an additional back-up annotator. During the annotation task, each of the three annotators received a stipulated hourly compensation of 2.56 times the minimum hourly wage in the Republic of Ireland.

### Annotation tool

To aid the annotation process, we utilise AWOCATo (Daudert, 2020), a custom-built annotation tool based on CoSACT (Daudert et al., 2019), the tool utilised to annotate the SemEval Task 5 Financial dataset (Cortis et al., 2017). It employs a continuous sentiment scale ranging from − 1 to 1, with 0 as neutral, as well as a highlight option to select the text portions expressing the respective sentiment. AWOCATo includes a relevancy scale to determine if a given text (i.e. microblog, news, or report) is relevant for a determined target. The annotation for the relevance follows the same scheme as for the sentiment, utilising a scale (ranging from 0 to 1). If the annotator is not able to provide an annotation, for example, due to insufficient knowledge, they can select the “I don’t know” option. In addition, the annotation tool includes a consolidation mode to enable additional input from a consolidator when the discrepancy among annotators for an annotation exceeds a predefined threshold. The interface of this tool in the consolidation mode is shown in Fig. 6; during the annotation phase, the table above “Submit” is not shown, the text is not pre-highlighted, and the scales default is set to 0.

During the annotation period, the selected annotators were invited to attend ten sessions with a duration of three hours to avoid exhaustion and jeopardising the annotations. To ensure constant concentration levels, the annotators could take breaks or finish the annotation session earlier. All these sessions had the author present to elucidate on any given question. Another five sessions occurred at home, without the author’s presence, as requested by the annotators and given the flexibility of the online-based annotation tool as accessible from any computer.

## Corpus statistics

**Fig. 7:**
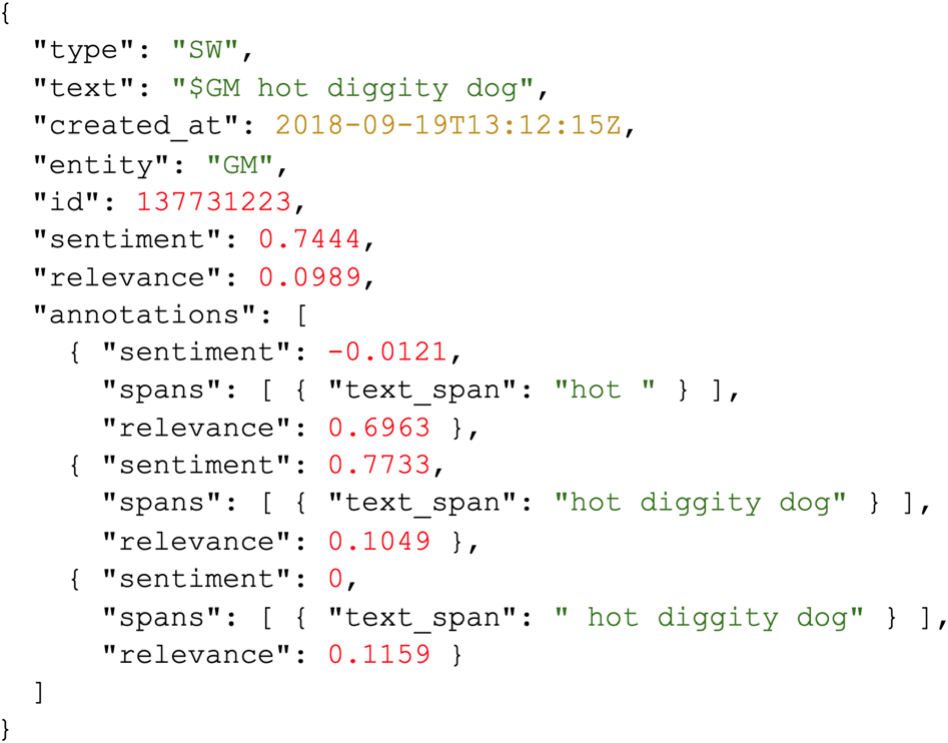
Data format example of an annotated stocktwit. Each annotation is provided individually so that researchers can analyse, for example, spans with their corresponding sentiment scores

The FinLin corpus contains a total of 3811 texts: 3204 stocktwits, 394 news articles, 127 company reports, and 86 investor reports. Figure 7 shows the data format example of an annotated stocktwit.

Table 2 shows the corpus statistics regarding the characters, words, and the annotated sentiment spans. As shown in Figs. 4 and 5, stocktwits provide the majority of the data followed by news articles, company reports and investor reports. StockTwits emphasises information sharing among its users, which occurs daily on multiple occasions; news also occur daily, thus, these are the major contributors for the FinLin corpus. In contrast, company reports occur quarterly. Investor reports can be published roughly twice-yearly per investor or randomly, focusing on event-based reporting e.g. when the company reports a profit warning. Thus, the data distribution corresponded to our expectations. Besides the frequency and number of observations, Fig. 5 also shows an irregular cyclic pattern for the stocktwits with a clear peak on July 25. As visible in Fig. 1 and 2, the most discussed companies where General Motors (GM) and Fiat-Chrysler (FCAU), thus, the peak in stocktwits can be explained by the United States imposed tariffs on steel which affected GM and the departure of Fiat-Chrysler’s CEO.

Overall, Ford, GM, and Fiat-Chrysler are the most covered entities, followed by Toyota Motor (TM) and Volkswagen (VLKAY), as present in Table 3. StockTwits is based in the United States of America (USA) where it is a known trading and information-sharing platform. Similarly, Ford, GM, and Fiat-Chrysler are also based in the USA which can indicate why these companies receive more attention than the remaining. The least covered entities are the French companies, Peugeot and Renault. As we collected reports, news, and stocktwits only in English, we believe that the little reporting on these targets, as well as low interest from USA users are the reason for the low coverage.

**Table 2:** Number of words and characters per text per data source, as well as the word count for the positive, neutral, and negative spans

Source	Words	Characters	Positive span # words	Negative span # words	Neutral span # words
Company reports	401	2,016	196.77	24.84	2.24
Investor reports	2,300	12,029	22.67	20.95	2.08
News articles	711	4,037	18.34	7.17	7.75
Stocktwits	24	109	4.39	4.01	2.48

All columns display average values. The symbol # represents “number of”

### Annotator performance

**Fig. 8:**
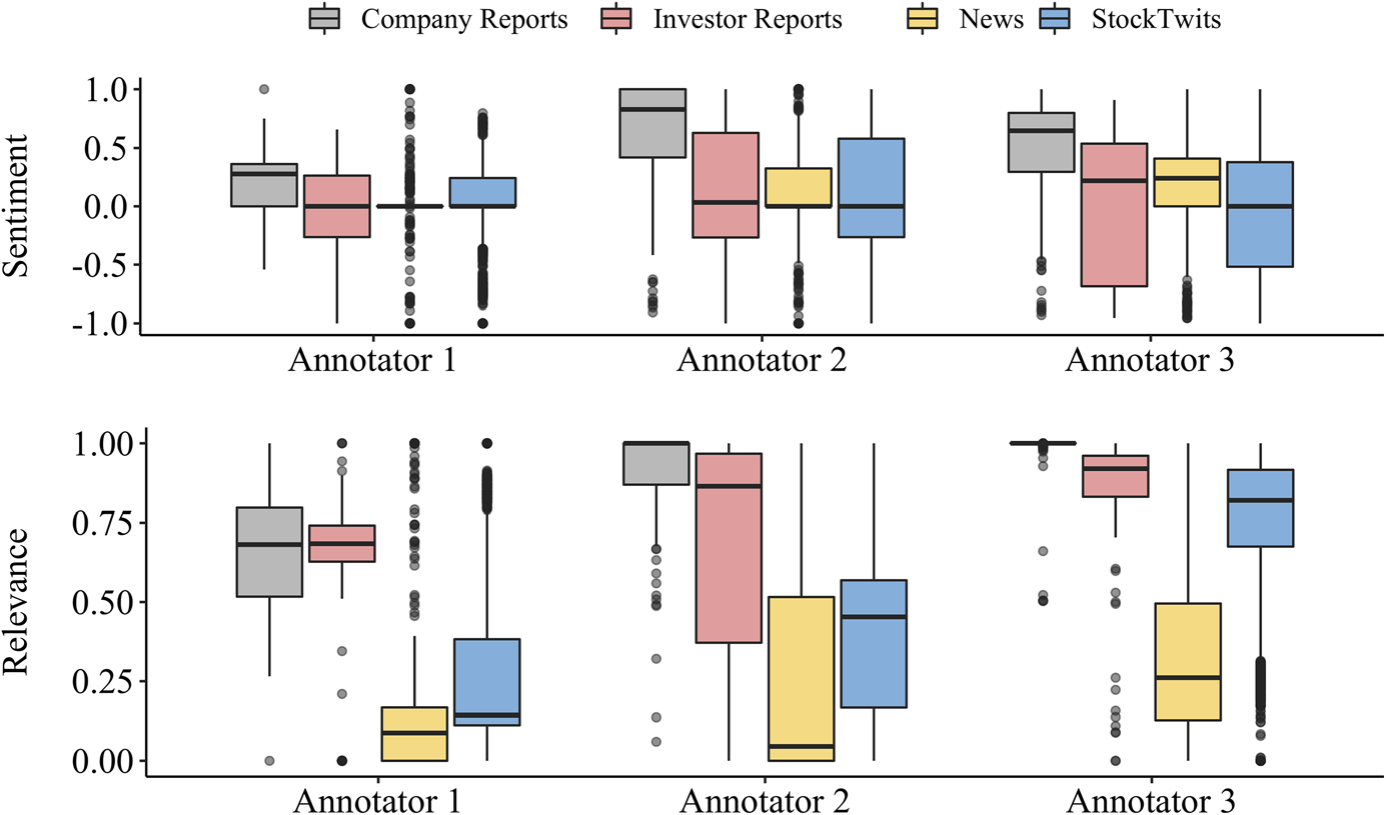
Box Plot for the sentiment and relevance annotation per annotator

The annotators’ performance is reported in Fig. 8. Overall, the sentiment annotation distribution was comparable among the annotators, with the exception of Annotator 1 in the company reports. In contrast, relevance annotation was far more heterogeneous which we attribute to the task difficulty, mainly due to the *point of view* interpretation. For example, the text “Mercedes-Benz USA Announces Senior Management Appointments” had one of the highest discrepancies; annotators 1 and 3 scored the relevance close to zero, and annotator 2 as one. Given that the annotation is performed on the entity Daimler, annotators 1 and 3 might have seen the new appointments, in the USA branch, of less importance to Daimler while annotator 2 potentially interpreted that senior management changes are relevant to the company. The inter-annotator agreement (IAA) was calculated utilising Fleiss’ Kappa (Fleiss, 1971). Given that all annotators annotated the same texts, we calculate the IAA on all texts. We report on these results for the relevance and sentiment on Table 4. Furthermore, we calculate and present in Table 5 the pair-wise IAA for the relevance and sentiment on each data type. As the annotation process was performed on a continuous scale, we transformed the sentiment scores into the polarity values (positive > 0, negative < 0, neutral = 0) and the relevance into three categories: low-relevance [0.0,0.25], medium-relevance [0.25,0.75], and high-relevance [0.75,1.0].

**Table 3:** Total number of occurrences per entity and source

	Company reports	Investor reports	News	Stocktwits	Total
BMWYY	5	4	9	55	73
DDAIF	16	8	24	7	55
F	32	32	96	700	860
FCAU	1	10	31	700	742
GM	5	16	32	700	753
HMC	1	0	34	234	269
HYMLF	0	4	38	0	42
NAV	9	1	0	200	210
NSANY	10	2	25	36	73
PEUGF	0	1	2	0	3
RNSDF	0	0	1	0	1
TM	7	3	48	345	403
VLKAY	41	5	54	227	327
Total	127	86	394	3204	3811

The IAA for the relevance annotation varies between *slight* for stocktwits and *substantial* for the investor reports according to Landis and Koch (1977) strength of agreement. Comparing the IAA with Fig. 8, annotation trends for each of the three annotators are visible; overall, the distribution (*i.e.* position of the box) of relevance is higher for the investor reports and lower across all annotators for the news, which reach the second-highest IAA. While the IAA for the news is *moderate*, it is *fair* for the company reports.

**Table 4:** Inter-annotator agreement (IAA) for each data source, reported in Fleiss’ Kappa

	Company reports	Investor reports	News	Stocktwits	Overall
Relevance IAA	0.3298	0.6477	0.4288	0.1219	0.1807
Sentiment IAA	0.4202	0.4663	0.3719	0.5953	0.5610

Comparing the IAA score for the relevance and sentiment, it is clear that the first task poses a higher difficulty than the latter. The IAA is *moderate* for the company reports, and investor reports, *fair-moderate* for the news, and *moderate-substantial* for the stocktwits. Furthermore, we observe clear differences between annotator 3 and the remaining, specifically for the relevance annotation in the company reports and stocktwits; this is also visible in Fig. 8. We believe this occurs due to the difficulty in defining and applying the point of view. As an example, consider the following text: “Glad $F is such a mess! Just keep screwing up new models.” It is clear what sentiment is portrayed (subjective: positive, factual: negative), however, the relevance relates to the point of view. If the point of view is targeting the author then the text is not relevant (i.e. they are a regular person) but if the point of view is targeting the company the text becomes highly relevant (*i.e.* the new car model seems to have issues which affects the future performance of the company).

In addition, Fig. 8 shows that annotator 1 refrains from extreme ratings, contrary to annotator 2 and 3, especially regarding the company reports. Given that we classify the relevance annotations into low, medium, and high, annotator 1 leads to disagreement as there is a tendency towards medium-relevance whereas the others tend to high-relevance. The same behaviour occurs for the sentiment annotations for which annotator 1 noticeably achieves the smallest distributions.

**Table 5:** Pair-wise IAA for each data source

		Company reports	Investor reports	News	Stocktwits
Relevance	Annotator 1 & 2	0.6632	0.7699	0.633	0.2603
	Annotator 1 & 3	0	0.6536	0.2946	0.0279
	Annotator 2 & 3	0	0.4963	0.398	0.0694
Sentiment	Annotator 1 & 2	0.4358	0.4302	0.4587	0.6177
	Annotator 1 & 3	0.3449	0.375	0.2574	0.5413
	Annotator 2 & 3	0.4752	0.6178	0.4553	0.6286

### Consolidation

Having three annotations per text in place, the FinLin dataset was consolidated either automatically or manually by an additional annotator (i.e. consolidator). The consolidation was conducted with AWOCATo, the same tool used for annotation. Manual consolidation occurred when (1) at least one of the annotations had a difference higher than the 3rd quantil to the mean of all annotations, or (2) when at least one of the annotators was not able to annotate a given text, or (3) when at least one of the annotations has a different polarity than the remaining. These thresholds are defined in Table 6 as well as the number of automatically and manually consolidated items.

**Table 6:** Thresholds implemented for consolidation, when the value to the mean exceeds the stipulated threshold manual consolidation is used, otherwise the annotations are automatically consolidated

	Company reports	Investor reports	News	Stocktwits
Relevance threshold	0.2	0.18	0.13	0.31
Sentiment threshold	0.31	0.3	0.21	0.26
Automatically consolidated	51	37	111	1,458
Manually consolidated	76 (59.84%)	49 (56.98%)	286 (72.59%)	1,746 (54.49%)

The thresholds were chosen based on the 3rd quantil distribution for the relevance and sentiment annotations

The consolidator’s aim was to select the final relevance and sentiment annotation while adhering to the mentioned annotation guidelines. The consolidator did not create an independent label; for a given annotation he utilised the lowest score as lower boundary and the highest score as the upper boundary, thus, serving as a mediator between the initial annotations. However, he also acted as a proofreader for cases in which one or two annotations were misplaced, for example when the text was misinterpreted. As shown in Fig. 6, the annotator was presented with the initial annotations, the text spans marked according to the annotators’ selections, and the scales default set to the mean relevance and mean sentiment scores of the initial annotations.

**Fig. 9:**
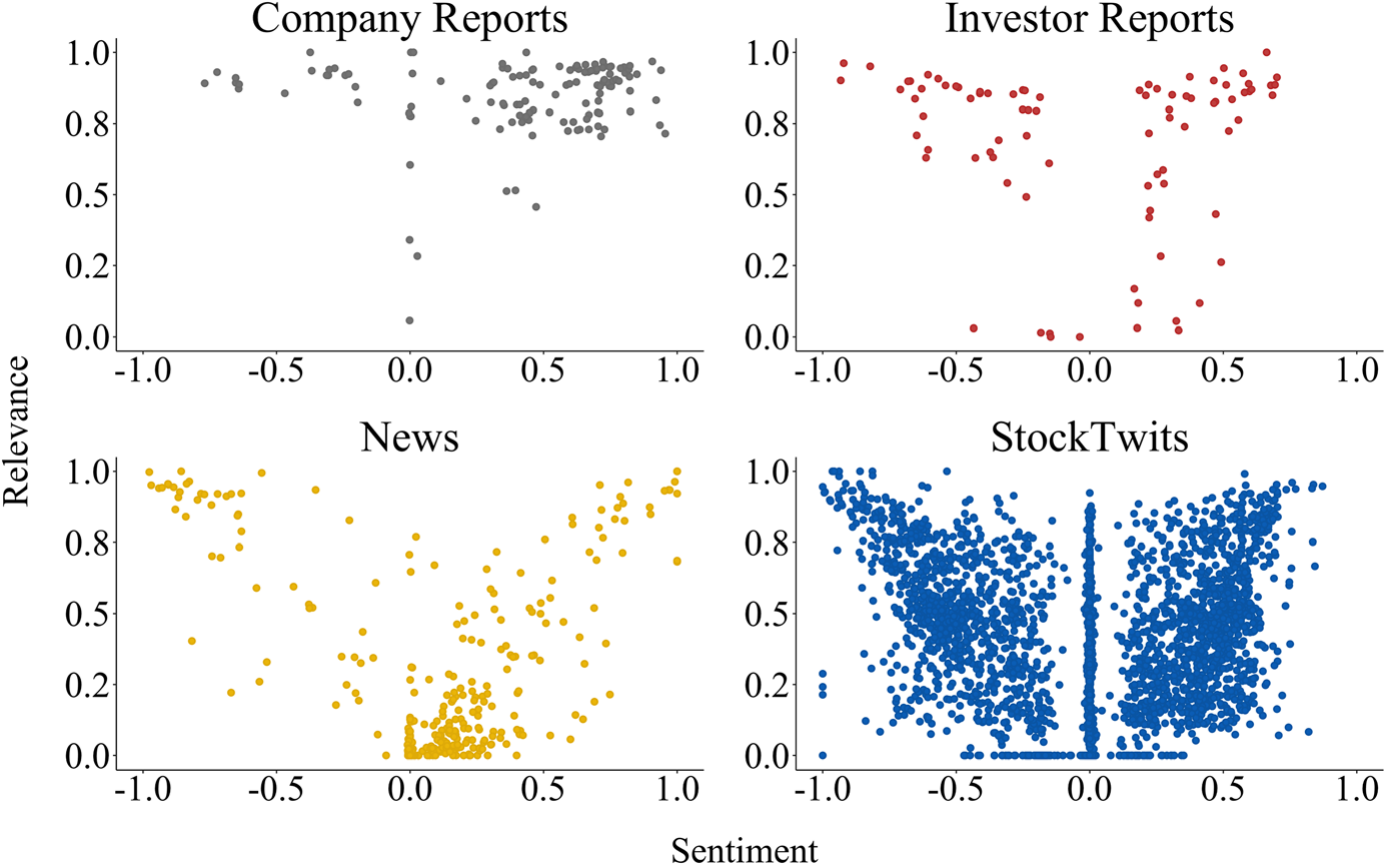
Distribution of the consolidated sentiment and relevance per data source

The final distribution of relevance and sentiment consolidations is shown in Fig. 9. The majority of the investor reports and company reports tend towards high relevance while news tend to have a lower relevance. In contrast, the stocktwits distribution is sparse, no clear tendency is observed. On one hand, this fits the nature of the investor and company reports, providing first-hand information about the targets’ business and performance or detailed (semi-)professional analyses. On the other hand, the low relevance of the news represents the propensity to summarize information and repurpose content (i.e. *news recycling*). Stocktwits are multifaceted, they can provide short analyses, first-hand/breaking news information, or promote the opinion of investors who can have an impact with their decisions.

Regarding the sentiment distribution, we can see that company reports are mainly positive. We hypothesize this is related to the marketing strategy employed by companies to ensure positive reporting, even when the results are poor. Comparing this with Table 3, we can observe that VLKAY (N = 41), F (N = 32), and DDAIF (N = 16) were responsible for the largest amount of the considered company reports. Given the prevailing topic of the *Diesel scandal* concerning Volkswagen, a cut in the profit forecast for Ford, and a change of the chief executive officer (CEO) regarding Daimler, company reports might be seen as an instrument to improve the public perception.

To a lesser extent, the news are also mainly positive. We can further observe that highly relevant news are either very negative or very positive, hence, we can assume these potentially contain important and novel information concerning a target. In contrast, news showing a low positive sentiment and relevance (the majority) are likely dealing with daily, trivial events in a non-polarising way. The number of published news articles, as shown in Table 3, also indicates public attention on Ford and Volkswagen (N = 96, N = 54).

Overall, the stocktwits distribution contrasts with the remaining sources, it is marked by low relevance with both positive and negative sentiment or neutral sentiment with varying relevance. Moreover, we can observe a rather symmetric distribution of positive and negative sentiment. Stocktwits’ short length limits the users’ opportunity to thoroughly explain a situation or carefully express a point of view. Contrary to news and reports, StockTwits represent a cross-sectional source. Stocktwits have a broad variety of users with distinct backgrounds and interests, for example, an investor might post a message similar to what one would find in a report, a non-expert user might post similarly to what one would find in a news article or could just express a random thought. This provides reasoning behind the distribution of the stocktwits annotations and the difficulty in its characterisation.

## Concluding remarks

This paper introduces a novel financial corpus for sentiment analysis - the FinLin corpus. It covers four distinct sources representing three data types (reports, news articles, and microblogs). FinLin includes a combined 3811 texts from investor reports, company reports, news, and stocktwits gathered from 01/July/2018 to 30/September/2018 and targeting a predefined set of entities from the automobile sector. The respective data was annotated for sentiment and relevance by three domain experts and further consolidated by a fourth expert, achieving an overall inter-annotator agreement of 0.1807 for the relevance and 0.5610 for the sentiment.

FinLin’s contemporaneous data covering the same entities provide an important resource for textual sentiment analysis across data sources and types. Further, our corpus also has potential applications in behavioural science as it provides insights on the sentiment’s relationship (i.e. how sentiment in one source is affected by or affects sentiment in the remaining). A novelty introduced in FinLin’s design was the relevance annotation; this provides additional knowledge on a text’s relevance to a company. As current works in financial sentiment analysis simply aggregate sentiments from multiple texts (Antweiler & Frank 2004; Jin et al., 2019), we identified the need for another measure for quantifying a text’s influence on the market sentiment. The relevance can potentially improve sentiment analysis, as suggested by previous related work, and poses itself another research task.

During the development of FinLin we encountered a few challenges. The first difficulty relates to the identification of the *point of view*, as well as the difference between *subjective sentiment* and *factual sentiment*. The second challenge was posed by the difficulty for the relevance annotation. While differentiating between relevant and non-relevant was feasible, the challenge is posed by the correct and subjective interpretation of the magnitude of a text’s relevance for a given company. As this depends on the annotators’ interpretation as well as their background, relevance annotations can exhibit a high variation even among experts. Lastly, the nature of the data source also posed challenges, especially stocktwits for which the limited length can potentially impede the correct interpretation of the text.

Overall, FinLin aims to complement the current knowledge by providing a novel and publicly available financial sentiment dataset and foster research on the topic of financial sentiment analysis.

## Supplementary Information

Below is the link to the electronic supplementary material.

Supplementary file1 (PDF 76 KB)

## Data Availability

The dataset is available at https://github.com/TDaudert/FinLin.

## Footnotes

1 https://www.imdb.com/

2 https://www.rottentomatoes.com/

3 https://mpqa.cs.pitt.edu/opinionfinder/

4 http://sentiwordnet.isti.cnr.it/

5 http://www.finet.hk/mainsite/index.htm

6 http://www.wjh.harvard.edu/~inquirer/homecat.htm

7 http://www.oica.net/wp-content/uploads/World-Ranking-of-Manufacturers-1.pdf

8 https://stocktwits.com/

9 https://about.stocktwits.com/

10 https://api.stocktwits.com/developers/docs

11 https://news.yahoo.com/

12 https://seekingalpha.com/

13 https://pandas.pydata.org/

14 https://www.investopedia.com/terms/m/marketsentiment.asp
