# Supplementary material for: A multi-source entity-level sentiment corpus for the financial domain: the FinLin corpus
Source: Lang Resour Eval. 2021 Aug 16;56(1):333–56. doi: 10.1007/s10579-021-09555-3 (PMC8817059; doi:10.1007/s10579-021-09555-3)
Supplement: Supplementary file 1 — Supplementary file1 (PDF 76 KB) [file 10579_2021_9555_MOESM1_ESM.pdf]

Supplementary Information

Annotation Guidelines

To ensure the annotators correctly understand the annotation task and to guide them throughout the annotation, we created a set of guidelines. These were introduced and recurrently available to the annotators; additional graphical guidance was also provided. Furthermore, these were updated to include the annotators' suggestions. Below, we detail each segment present in the annotation guideline.

Goals

1. Determine how relevant the text displayed is to a presented company name.
2. In case it is relevant, determine what is the sentiment of the message.
3. Mark parts of the text which clearly reflect the sentiment.

Definitions

In this context, *relevant* means a text containing information about or related to the company name given (*e.g.* Ford). Depending on the importance of the mentioned event on the company's business, the information stated in the text can be more or less relevant to the company. In the case of a completely non-relevant text, the annotators are asked to just click "Submit" without moving the relevance slider. In all other cases, the annotators adjust the slider the more to the right the more relevant the given information is for the presented company. Examples for the company Ford are:

1. "Ford's profit increase by 20% in 2017" → relevant
2. "Ford is teaming up with Tesla for a new collaboration" → relevant
3. "Ford's CEO got injured in a car crash" → relevant
4. "Harrison Ford is the best actor of the year" → not relevant

As seen, all relevant examples refer to Ford as a company. However, they are not all referring to the same aspect of its business. The annotators task is to annotate with higher relevance, the text which, in their opinion, is more relevant for a company's business.

In the economic context, the *sentiment* of a news text reflects on the company's situation or outlook in the future. Since some authors aim at providing an objective piece of text, one might not be able to understand whether the author thinks the event/fact they are reporting on is good or bad for a company. Therefore, the annotators task is to judge the presented information. A text's sentiment towards a company, an associated entity, or an event can range from positive (bullish) to negative (bearish) with neutral sentiment in between. This is represented by a slider which is neutral on default. The slider is only moved in cases where the sentiment is not neutral. It is moved the more to the edges of the scale the more a text is positive or negative. A text is more positive (or negative), the more impactful a presented event/fact is for a company, or the stronger the language used by the author. This will depend on the annotators interpretation of the given information. Furthermore, the annotators are asked to relatively judge the sentiment based on information the have already annotated. Examples for the company Ford are:

1. "Ford is teaming up with Tesla for a new collaboration"$\rightarrow$ positive
2. "Ford's CEO got injured in a car crash" $\rightarrow$ negative
3. "Ford should buy Tesla" $\rightarrow$ neutral
4. "Ford is releasing its new business plan" $\rightarrow$ neutral

Sometimes, specific parts (*i.e.* span) of the text clearly reflect the message's sentiment; while other parts of the text are less informative. If this is the case in the text displayed, the annotators highlight the identified span. Examples for the company Ford are: "Ford's **profit increase by 20% in 2017**", "Ford is **teaming up with Tesla for a new collaboration**", "Ford **will release a new model** at the end of this year", "Ford's **CEO got injured** in a car crash", "Ford's **business seems to be shaky**", "Ford **should buy Tesla**", "Ford **is releasing its new business plan**".
